# Consumption of Energy Drinks and Use of Dietary Supplements Among Italian University Students: A Cross-Sectional Study

**DOI:** 10.3390/nu18203386

**Published:** 2026-10-15

**Authors:** Federica Vaccaro, Giuseppe Di Martino, Pamela Di Giovanni, Tommaso Staniscia

**Affiliations:** 1 Department of Medicine and Ageing Sciences, “G. d’Annunzio” University of Chieti-Pescara, 66100 Chieti, Italy; giuseppe.dimartino@unich.it (G.D.M.); pamela.digiovanni@unich.it (P.D.G.); tommaso.staniscia@unich.it (T.S.); 2 Unit of Epidemiology and Health Statistic, Local Health Authority of Pescara, 65100 Pescara, Italy

**Keywords:** energy drinks, dietary supplements, university students, physical activity

## Abstract

Background/Objectives: The rising consumption of energy drinks (ED) and dietary supplements (DS) among youth raises public health concerns, particularly regarding uncontrolled use, excessive dosages, and product combinations. This study assessed ED and DS use among a sample of university students in Southern Italy and identified associations with sociodemographic and lifestyle characteristics. Methods: Students from “G. d’Annunzio” University of Chieti-Pescara completed a web-based questionnaire on socio-demographic characteristics, ED and DS consumption, and health-related behaviors between February and September 2023. Results: A total of 404 students participated (74.8% female; mean age 22.1 ± 3.9 years). Overall, 27.9% reported ED consumption, and 36.7% regularly used DS, primarily proteins and amino acids (33.8%). Regarding lifestyle, 45.3% were smokers, and 68.3% did not engage in regular physical activity. Multivariable analysis showed that ED consumption was positively associated with male sex (OR 3.63; 95% CI 1.94–6.79) and team sports participation (OR 6.59; 95% CI 1.76–24.60). DS use was significantly higher among physically active students (*p* < 0.001) and was positively associated with weight training (OR 32.2; 95% CI 9.36–111.2) and aerobic exercise (OR 3.03; 95% CI 1.34–6.85), while being negatively associated with male sex (OR 0.50; 95% CI 0.29–0.87). Smoking status and BMI showed no significant associations with ED or DS use. Conclusions: These findings highlight a substantial prevalence of ED and DS use among university students enrolled in health-related courses, linked to specific sporting activities rather than general lifestyle risks. Targeted educational interventions may help promote informed and responsible consumption in this population.

## 1. Introduction

Over the past two decades, energy drinks (ED) and dietary supplements (DS) have become increasingly popular, particularly among young adults and university students. Despite their widespread availability in stores and online, they differ considerably in terms of ingredients, purpose and regulations [1,2].

Specifically for ED, market growth has been fueled by aggressive business expansion and advertising campaigns, particularly those targeting younger individuals [3,4]. ED, typically non-alcoholic beverages containing caffeine (50–400 mg/L), taurine, herbal extracts, and B-group vitamins, are marketed for their supposed ability to improve physical and mental performance [5]. DS, on the other hand, have become a common part of many people’s health routines [6,7], while still facing regulatory challenges. Within the European Union, legislation governing DS is only partially uniform—especially for botanical products—leading to differences in safety evaluations and labeling across countries [8,9]. This regulatory inconsistency, combined with free movement of goods and easy online access, encourages their widespread use even when there is no real medical need or professional advice involved [10].

Among university students, ED are often consumed to combat fatigue, improve alertness, and sustain attention during academic or physical activities [2,11]. Originally introduced in Asia in the 1960s and later spreading rapidly across Europe after 1987 [12], these beverages now represent a major global market, valued at USD 45.8 billion in 2020 and projected to exceed USD 108 billion by 2031 [13]. The appeal of ED is strongly tied to lifestyle factors and marketing strategies that associate their consumption with improved performance, vitality, and social identity [14,15]. However, frequent or excessive intake can lead to adverse effects such as insomnia, jitteriness, gastrointestinal discomfort, and increased blood pressure and heart rate, while more severe cases, including arrhythmias and hypertension, have also been reported [16,17,18,19].

A similar trend has been observed with DS, which are increasingly used for perceived benefits related to health, energy, and appearance [20]. Regulated as food products, DS are concentrated sources of nutrients or other substances with nutritional or physiological effects, and are available in dose forms such as tablets, capsules, or measured liquids [8]. They aim to address various needs, from correcting micronutrient deficiencies and supporting macronutrient intake to promoting overall health, well-being, and adaptation to physiological stress [21]. Their widespread use reflects a growing public awareness of health and disease prevention, as well as a desire to maintain well-being and manage age-related changes [22]. Among young adults, especially university students, supplement use goes beyond physical performance. Studies show that they are also used to support general health, boost energy, enhance appearance, or respond to specific lifestyle demands [23,24]. Despite their popularity, the efficacy and safety of DS remain debated, as supporting evidence is often inconsistent [25,26,27,28,29], and excessive or inappropriate use can pose health risks [30,31,32].

Investigating ED and DS together is warranted because university students often use both products as complementary strategies to manage their lifestyles, enhance performance, and cope with academic fatigue [33]. Although ED and DS are distinct products—with DS having a specific regulatory classification in Europe [8], whereas ED lack a formal legal category—their distinction may be less clear to young consumers because of overlapping functional claims and shared active ingredients. In this study, ED were defined as caffeinated functional beverages, while DS referred to concentrated products intended to supplement the regular diet.

ED consumption and DS use are often examined separately, and, to the best of our knowledge, no previous studies have examined both in a similar cohort. Evaluating these behaviors concurrently can help identify distinct but potentially interconnected patterns of use and highlight subgroups at greater risk of combined or excessive intake. We therefore hypothesized that, despite the functional overlap, ED consumption and DS use are distinct behavioral patterns, driven by different lifestyle motives and sociodemographic and behavioral predictors. Accordingly, this study aimed to assess the prevalence and lifestyle correlates of ED consumption and DS use among university students, both separately and concurrently.

## 2. Materials and Methods

The present study represents a secondary analysis of the CCAP study (Change in Caffeine Consumption after the Pandemic (CCAP Study) among University Students: A Cross-Sectional Study from Italy) [34]. Specifically, we collected no new data; instead, we reanalyzed the original dataset using a different analytical framework. While the original publication evaluated caffeine consumption trends across pandemic phases, the present study addresses a distinct research question by examining how ED and DS behaviors interact with sociodemographic and lifestyle determinants.

### 2.1. Study Design

All students attending the “G. d’Annunzio” University of Chieti-Pescara were eligible to participate. The questionnaire link was shared via social media and promoted directly to students during lectures. To enhance participation, enrolled students were also encouraged to share the survey with their peers. Participation was voluntary. Data collection took place between February and September 2023. The sample size calculation was described elsewhere [34] and indicated that a minimum of 378 students was required to meet the study objectives. However, because this investigation is a secondary analysis focusing on ED and DS consumption, no separate a priori sample size estimation was performed for this objective, and the full available dataset was analyzed.

### 2.2. Informed Consent and Ethical Approval

The questionnaire cover letter briefly described the aims of the study and informed participants of its scientific purpose. All collected data were downloaded and stored on a secure computer with password-protected access. Participants provided informed consent prior to participation.

The collected questionnaire data were completely anonymous, preventing any direct or indirect identification of respondents or posing any risk of harm. Data were analyzed and reported exclusively in aggregate form, in accordance with Italian and European regulations on personal data protection (DL 101/2018 and Regulation (EU) 2016/679, General Data Protection Regulation-GDPR) [21,22].

According to the internal regulations of the competent Ethics Committee, which served for the referral board of this study, formal ethical committee approval is explicitly exempted for non-pharmacological, observational studies based exclusively on fully anonymous, voluntary surveys that do not collect sensitive biological or identifiable personal data. Data were analyzed and reported solely in aggregate form.

### 2.3. Questionnaire

The original survey was organized into five main sections [34]. For the present study, the analysis was limited to Section 1, which comprised 10 questions on sociodemographic characteristics, and Section 3, which contained 12 questions on caffeine, ED consumption, and DS use patterns.

Data on DS use and ED consumption were collected using structured, closed-ended items. A 30-day recall period was used to assess active, regular exposure to both product categories. For DS, participants were asked whether they had used any DS during the reference period (yes/no). Participants reporting “yes” were categorized as DS users; “regular use” was defined as the intentional, self-administered intake of one DS over the past 30 days. Detailed closed-ended sub-questions were used to identify specific supplement types and primary motives for intake. For ED, consumption frequency was assessed using closed-ended ordinal categories (<1 time/week, 1–3 times/week, and >3 times/week). All survey items were mandatory, resulting in a complete dataset with no missing values.

### 2.4. Statistical Analysis

Continuous variables (BMI and age) were assessed for normality using the Kolmogorov–Smirnov test and, as they were normally distributed, were expressed as mean ± standard deviation. Categorical variables were reported as frequencies and percentages. Group comparisons for categorical variables were performed using Pearson’s chi-squared test or Fisher’s exact test, as appropriate.

Multivariable logistic regression models were conducted to evaluate factors associated with ED and DS consumption. Models were fully adjusted, and multicollinearity was assessed using the Variance Inflation Factor (VIF), with all variables demonstrating a VIF < 4. Model fit and explanatory power were evaluated using Nagelkerke’s R^2^. To address potential sparse-data bias or quasi-complete separation, a sensitivity analysis was conducted using Firth’s penalized likelihood logistic regression.

Due to the low number of ED and DS co-users, multivariable logistic regression could not be performed for this subgroup, and only univariate analyses were reported.

Given the cross-sectional design and secondary nature of the study, all statistical analyses were considered exploratory, and multiple comparisons were interpreted cautiously.

A *p*-value < 0.05 was considered statistically significant. All analyses were performed using STATA version 14 (StataCorp LLC, College Station, TX, USA).

## 3. Results

The study population consisted of 404 students, predominantly female and enrolled in Health Professions degree programs (Table 1). Regarding lifestyle habits, daily coffee consumption (*n* = 313; 77.5%) and smoking were common across the sample. Conversely, physical activity levels were generally low, with fewer than one-third of students engaging in regular exercise. Among those who did, weight training was the most common discipline (Table 1).

Overall, more than a third of participants reported regular use of DS, driven primarily by performance enhancement (Table 2). Supplement choices varied significantly by physical activity type (*p* < 0.001), with weightlifters mostly using protein and aerobic exercisers opting for carbohydrates. ED use was less frequent, mostly occurring occasionally (less than once per week), whereas concurrent use of ED and DS was rare (4.2%; Table 2).

Bivariate analysis showed that DS use was significantly more common among physically active participants than among inactive peers (*p* < 0.001; Table 3), whereas no significant association was observed between physical activity and ED consumption (*p* = 0.962). Additionally, both active and inactive participants rarely reported using ED and DS concurrently (Table 3).

As shown in Table 4, multivariable logistic regression analysis revealed that ED consumption was significantly associated with male sex (OR 3.63, *p* < 0.001), higher coffee intake (OR 1.28, *p* = 0.031), and sports team participation (OR 6.59, *p* = 0.005). Conversely, DS use was strongly associated with physical activity patterns, particularly weight training (OR 32.2, *p* < 0.001) and aerobic exercise (OR 3.03, *p* < 0.001), and was negatively associated with male sex (OR 0.5, *p* = 0.015). Other variables, including age, smoking status, and BMI, showed no significant association with use of either product. Sensitivity analysis using Firth’s penalized logistic regression confirmed both the direction and statistical significance of these findings (Table S1).

As reported in Table 5, only the age variable was significantly associated with co-use; however, this result was obtained using a univariate model.

## 4. Discussion

The present study provides a comprehensive overview of ED and DS consumption among a sample of university students in Southern Italy, mostly enrolled in health-related degree programs.

A substantial proportion of students (27.9%, *n* = 113) reported consuming ED, consistent with findings from previous national and international studies [35,36,37,38,39]. Among consumers, the frequency of intake was concentrated in the lowest consumption category, with 64.6% (*n* = 73) consuming ED less than once a week.

Despite this low consumption frequency, these findings highlight that ED remains a notable component of students’ dietary behaviors, particularly amid academic stress and lifestyle patterns commonly observed in university populations [40,41].

Consistent with previous findings, male gender was independently associated with a higher likelihood of ED consumption (OR = 3.63, 95% CI [2.22–5.94]) [42,43,44,45] and was associated with higher daily coffee intake, supporting evidence that ED users tend to have greater overall caffeine consumption [46,47]. Although bivariate analysis did not show a significant association between overall physical activity and ED use, multivariable logistic regression identified a more specific pattern. After accounting for potential confounders, participation in a sports team emerged as an independent factor associated with higher ED consumption, whereas general physical activity did not. This suggests that it is not being physically active per se that is linked to ED use, but rather engagement in organized, competitive sport contexts, where performance pressure, team culture, and targeted marketing may normalize or even encourage ED intake. This pattern is consistent with previous research in university and adolescent populations, which has shown higher ED consumption among team sport athletes compared with non-athletes or recreationally active peers [48,49,50].

Notably, despite their health-focused academic background, nearly half of the participants were smokers (45.3%), and approximately two-thirds led a sedentary lifestyle. This high prevalence of smoking among future healthcare professionals aligns with previous international literature, which suggests that health-related academic curricula do not automatically translate into healthier personal lifestyle choices [12,35,50,51].

Regarding DS, 36.7% (*n* = 148) of students reported their use. In contrast to the pattern observed for ED, DS use showed a clear association with physical activity (*p* < 0.001), in both bivariate analysis and multivariable models, with Firth’s regression confirming the robustness of these findings; notably, this association extended across different types of physical activity.

This finding is consistent with a large body of research showing that physically active students are more likely to use DS to meet increased nutritional demands, enhance performance, or support recovery [43,52,53,54]. Such behavior is often interpreted as part of a proactive, health- and fitness-oriented profile, where regular exercise drives the search for functional nutritional support [22,23].

The high prevalence of protein and amino acid supplement use (33.8%, *n* = 50) in our sample is consistent with this profile, as these products are among the most consumed by students and athletes seeking to build or maintain muscle mass and to enhance performance or recovery [23,43,53,55]. The lower odds of DS use observed among male participants (OR = 0.5, 95% CI [0.28–0.87]) may point to sex-specific differences in health patterns, which might align with a multifaceted health orientation among female students. This pattern is consistent with previous studies indicating that women more frequently report using multivitamins and micronutrient-focused supplements, as well as greater overall interest in health-promoting dietary behaviors, even in contexts where the overall prevalence of supplement use is comparable to or higher among men [22,24,56,57,58]. However, these behavioral drivers were not directly measured in our dataset and should be formally tested in future research.

Finally, in the univariate analysis of concurrent ED and DS use, younger age was the only factor associated with a higher likelihood of co-use (OR = 0.52, 95% CI [0.33–0.84]). Gender, BMI, smoking, coffee consumption, and physical activity modalities showed no significant associations. Nevertheless, the low prevalence of co-use, together with the absence of shared multivariable predictors, suggests that ED consumption and DS use in this university population are distinct behavioral practices, driven by different motivations (e.g., managing acute academic fatigue versus routine fitness and health maintenance), rather than a clustered poly-substance behavior.

This study has several limitations that should be considered when interpreting the findings. First, its cross-sectional design precludes any inference of causal relationships between lifestyle factors and the use of ED or DS. Second, all data were self-reported and may therefore be subject to recall and social desirability bias. Furthermore, although the questionnaire was based on the available literature and designed to include clear, straightforward items, it was not formally validated. Third, because this was a secondary analysis of an existing dataset, an a priori sample size calculation was not specifically performed for ED and DS outcomes, which may limit statistical power to detect subtle associations in smaller subgroups.

Moreover, the study relied on a nonprobability convenience sampling strategy at a single university in Southern Italy. Because the exact denominator of students reached could not be determined, a formal response rate could not be calculated, introducing potential selection bias.

Furthermore, female students and those enrolled in health-related disciplines were overrepresented, which limits the generalizability of our findings to the broader university population. Finally, owing to sparse data in specific subgroups (such as participants involved in competitive sports teams), some odds ratios displayed wide confidence intervals, indicating that these estimates should be interpreted with caution and confirmed in larger samples.

## 5. Conclusions

This study shows a substantial prevalence of ED and DS use among university students attending a single institution in Southern Italy enrolled in health-related degree programs. ED consumption was predominantly associated with male sex, higher caffeine intake, and participation in sports teams, whereas DS use was more strongly linked to fitness-oriented physical activity behaviors. Overall, these findings suggest the potential utility of targeted, university-based educational interventions to promote informed and responsible use of these products among future healthcare professionals, although findings regarding specific activity subgroups warrant confirmation in larger student populations.

## Figures and Tables

**Table 1:** Baseline characteristics of the Study Population.

	N	%
**Age** (*mean ± SD*)	22.1 ± 3.9	
**Gender**		
F	302	74.8
M	102	25.2
**BMI**		
Underweight (<18.5)	3	0.7
Normal weight (18.5–24.9)	295	73.1
Overweight (25.0–29.9)	98	24.2
Obese (≥30.0)	8	2.0
**Degree course**		
Health Professions	243	60.2
Medicine and Dentistry	48	11.9
Pharmacy	99	24.5
Food science	10	2.5
Non-health related	4	0.9
**Geographic Region of Origin**		
Central Italy	23	5.7
Southern Italy	377	93.3
Northern Italy	1	0.2
Foreign Country	3	0.8
**Smoking status**		
Non-smoker	221	54.7
Smoker	183	45.3
**Coffee per day**		
None	91	22.5
1–2	112	27.8
3–4	137	33.9
>5	64	15.8
**Physical activity**		
No	276	68.3
Yes	128	31.7
**Frequency of physical activity**		
1–2 times/week	17	13.3
3–4 times/week	95	74.2
>4 times/week	16	12.5
**Types of physical activity**		
Weight training	76	59.4
Aerobic training	33	25.8
Sports team	11	8.5
Other	8	6.3

SD: standard deviation; BMI: Body Mass Index.

**Table 2:** DS and ED: patterns of consumption and use.

	N	%
**DS use**		
Yes	148	36.7
No	256	63.3
**Type of DS**		
Proteins/amino acids	50	33.8
Vitamins and minerals	38	25.6
Carbohydrate-based	28	18.9
Probiotics	20	13.5
Immune boosting	10	6.8
Fatty acids	2	1.4
**Reason for DS use**		
Improve performance	82	55.4
Improve overall health	52	35.2
Prevent diseases	12	8.1
Clinical reasons	2	1.3
**ED consumption**		
Yes	113	27.9
No	291	72.1
**Frequency of ED use**		
Less than once/week	73	64.6
1–3 times/week	29	25.7
More than 3 times/week	11	9.7
**ED + DS co-use**	17	4.2

ED: energy drinks; DS: dietary supplements.

**Table 3:** Bivariate analysis of ED consumption, DS use and ED + DS co-use according to physical activity.

		**DS Use**		
		**N (%)**		
		**Yes**	**No**	**Total**
Physical activity	Yes	79 (61.7)	49 (38.3)	128
	No	69 (25.0)	207 (75.0)	276
	Total	148	256	404
*p* < 0.001				
		**ED consumption**		
		**N (%)**		
		**Yes**	**No**	**Total**
Physical activity	Yes	36 (28.1)	92 (71.9)	128
	No	77 (27.9)	199 (72.1)	276
	Total	113	291	404
		**ED and DS co-use**		
		**N (%)**		
		**Yes**	**No**	**Total**
Physical activity	Yes	9 (7.1)	119 (92.9)	128
	No	8 (2.9)	268 (97.1)	276
	Total	17	387	404
*p* = 0.054				

ED: energy drinks; DS: dietary supplements.

**Table 4:** Multivariable logistic regression analysis of factors associated with ED and DS consumption.

	ED Consumption			DS Use		
	OR	*p*	95% CI	OR	*p*	95% CI
Male gender	3.63	<0.001	2.22–5.94	0.5	0.015	0.28–0.87
Age	0.99	0.928	0.93–1.06	1.01	0.506	0.96–1.07
Coffee	1.28	0.031	1.02–1.61	1.21	0.093	0.96–1.53
Smoking	1.04	0.861	0.65–1.67	1.05	0.817	0.66–1.67
BMI	0.92	0.123	0.84–1.02	0.97	0.539	0.88–1.06
Physical activity						
None (Ref.)	1					
Weight training	0.73	0.496	0.30–1.78	32.2	<0.001	9.36–111.2
Aerobic training	0.86	0.642	0.47–1.59	3.03	<0.001	1.77–5.15
Sports team	6.59	0.005	1.79–24.2	7.7	0.004	1.93–30.67
Other	0.85	0.857	0.12–4.64	1.45	0.621	0.33–6.37

ED: energy drinks; DS: dietary supplements; OR: Odds Ratio; CI: Confidence Interval; BMI: Body Mass Index. Age, coffee consumption (cups/day) and BMI (kg/m^2^) were entered into the multivariable model as continuous variables. Physical activity categories were mutually exclusive based on the primary type of exercise. Reference categories: Female gender; Non-smoker; No physical activity.

**Table 5:** Univariate logistic regression analysis of factors associated with ED and DS co-use.

	ED + DS Co-Use		
	OR	*p*	95% CI
Male gender	0.62	0.465	0.18–2.21
Age	0.52	0.007	0.33–0.84
Coffee	1.10	0.680	0.68–1.80
Smoking	0.65	0.401	0.23–1.79
BMI	1.00	0.979	0.83–1.21
Physical activity			
None (Ref.)	1		
Weight training	2.16	0.343	0.44–10.64
Aerobic training	2.87	0.058	0.96–8.55
Sports team	3.35	0.275	0.38–29.42

ED: energy drinks; DS: dietary supplements; OR: Odds Ratio; CI: Confidence Interval; BMI: Body Mass Index. Age, coffee consumption (cups/day) and BMI (kg/m^2^) were entered into the multivariable model as continuous variables. Physical activity categories were mutually exclusive based on the primary type of exercise. Reference categories: Female gender; Non-smoker; No physical activity.

## Data Availability

Data was available after reasonable request due to privacy restriction.

## Supplementary Materials

The following supporting information can be downloaded at: https://www.mdpi.com/article/10.3390/nu18203386/s1, Table S1: Multivariable Firth’s penalized logistic regression analysis of factors associated with ED and DS consumption.
