# Supplementary material for: Consumption of Energy Drinks and Use of Dietary Supplements Among Italian University Students: A Cross-Sectional Study
Source: Nutrients. 2026 Oct 15;18(20):3386. doi: 10.3390/nu18203386 (PMC13708152; doi:10.3390/nu18203386)
Supplement: Supplementary file 1 [file nutrients-18-03386-s001.zip › nutrients-4568560-supplementary.pdf]

Table S1. Multivariable Firth's penalized logistic regression analysis of factors associated with ED and DS consumption

	ED consumption			DS use		
	OR	p	95% CI	OR	p	95% CI
Male gender	1.30	0.049	1.00-3.19	0.6	0.076	0.34-1.05
Age	0.81	0.026	0.67-0.97	1.03	0.237	0.98-1.09
Coffee	1.50	0.053	0.99-2.29	1.95	<0.001	1.40-2.74
Smoking	1.16	0.600	0.65-2.09	1.07	0.784	0.67-1.70
BMI	0.94	0.345	0.83-1.06	0.98	0.620	0.89-1.07
Physical activity						
None (Ref.)	1					
Weight training	0.78	0.658	0.27-2.30	25.7	< 0.001	8.04-82.48
Aerobic training	0.85	0.674	0.39-1.18	2.94	< 0.001	1.73-5.03
Sports team	0.82	0.835	0.14-4.87	6.6	0.004	1.81-24.09
Other	2.45	0.268	0.50-11.97	1.74	0.447	0.41-7.38

OR: Odds Ratio; CI: Confidence Interval; BMI: Body Mass Index.

Age, coffee consumption (cups/day) and BMI (kg/m^2) were entered into the multivariable model as continuous variables. Physical activity categories were mutually exclusive based on the primary type of exercise. Reference categories: Female gender; Non-smoker; No physical activity.
